# Development and Characterization of a *Psathyrostachys huashanica* Keng 7Ns Chromosome Addition Line with Leaf Rust Resistance

**DOI:** 10.1371/journal.pone.0070879

**Published:** 2013-08-19

**Authors:** Wanli Du, Jing Wang, Liangming Wang, Jun Zhang, Xinhong Chen, Jixin Zhao, Qunhui Yang, Jun Wu

**Affiliations:** Shaanxi Key Laboratory of Genetic Engineering for Plant Breeding, College of Agronomy, Northwest A&F University, Yangling, Shaanxi, China; United States Department of Agriculture, United States of America

## Abstract

The aim of this study was to characterize a *Triticum aestivum*-*Psathyrostachys huashanica* Keng (2n = 2x = 14, **NsNs**) disomic addition line 2-1-6-3. Individual line 2-1-6-3 plants were analyzed using cytological, genomic *in situ* hybridization (GISH), EST-SSR, and EST-STS techniques. The alien addition line 2-1-6-3 was shown to have two *P. huashanica* chromosomes, with a meiotic configuration of 2*n* = 44 = 22 II. We tested 55 EST-SSR and 336 EST-STS primer pairs that mapped onto seven different wheat chromosomes using DNA from parents and the *P. huashanica* addition line. One EST-SSR and nine EST-STS primer pairs indicated that the additional chromosome of *P. huashanica* belonged to homoeologous group 7, the diagnostic fragments of five EST-STS markers (*BE404955*, *BE591127*, *BE637663*, *BF482781* and *CD452422*) were cloned, sequenced and compared. The results showed that the amplified polymorphic bands of *P. huashanica* and disomic addition line 2-1-6-3 shared 100% sequence identity, which was designated as the 7**Ns** disomic addition line. Disomic addition line 2-1-6-3 was evaluated to test the leaf rust resistance of adult stages in the field. We found that one pair of the 7**Ns** genome chromosomes carried new leaf rust resistance gene(s). Moreover, wheat line 2-1-6-3 had a superior numbers of florets and grains per spike, which were associated with the introgression of the paired *P. huashanica* chromosomes. These high levels of disease resistance and stable, excellent agronomic traits suggest that this line could be utilized as a novel donor in wheat breeding programs.

## Introduction

The leaf rust caused by *Puccinia recondita* Roberge ex Desmaz. f. sp. *tritici* Eriks. & E. Henn leads to high annual losses because of its widespread occurrence, although leaf rusts generally produce fewer losses than stem rust and stripe rust. [1]. Over 50 leaf rust resistance (*Lr*) genes have been cataloged and mapped to specific chromosomes [2], almost half of which are derived from wheat relatives, including *Aegilops* [3], *Agropyron* [4], *Hordeum* [5], *Secale cereale* L. [6], *Thinopyrum* [7], and *Lophopyrum ponticum* [8]. Many different addition lines, substitution lines, translocation lines, and introgression lines have been developed in wheat, and their desirable traits have been characterized to facilitate the study of alien chromosomes or segments that carry excellent traits.

*Psathyrostachys* is a small genus containing no more than ten perennial species, which are distributed throughout central Asia, from east Turkey to central China and Mongolia. *Psathyrostachys* species are known to be diploid (2*n* = 14) with the basic genome **Ns** [9]. *Psathyrostachys huashanica* Keng (2*n* = 2*x* = 14, **NsNs**) is morphologically distinct from all other species in the genus and it is also geographically isolated because it grows only in a narrow area of the mountainous rocky slopes of Mount Huashan, Shaanxi Province, central China [10], [11], [12]. *P. huashanica* has attracted considerable attention from wheat breeders as an outcrossing material because of its early maturity and resistance to cold, drought, disease, barren soil, and salinity [13], [14], [15], [16], [17], [18], [19], [20].

Interspecific hybridization between common wheat and its wild relatives is an effective and economic method for introducing agronomically desirable characters into available wheat cultivars [21], [22]. The production of addition lines is an important step during the successful transfer of alien genes into wheat. In 1991, our research team successfully produced the hybrid H881 (2*n* = 28, **ABDNs**) from common wheat cultivar 7182 and *P. huashanica* via embryo culture, and backcrossing induced the spontaneous doubling of chromosomes, which generated the heptaploid hybrid H8911 (2*n* = 49, **AABBDDNs**) [23]. A batch of wheat-*P. huashanica* monosomic addition lines were then developed after a second backcrossing [24], followed by strict generations of selfing accompanied by cytology, genomic *in situ* hybridization (GISH) screening, molecular marker analysis, and morphological observation. We identified the BC_2_F_2_ line 2-1-6-3 as having a pair of 7**Ns** chromosomes. An evaluation of leaf rust resistance during the adult stages showed that this added pair of 7**Ns** chromosomes carried leaf rust resistance gene(s) in a wheat background, which may be useful for comparative research and for the exploitation of desirable *P. huashanica* genes in wheat improvement. The superior numbers of florets and grains per spike in wheat line 2-1-6-3 were also related to the introgression of the pair of *P. huashanica* chromosomes.

The objectives of this study were: a) to identify and characterize the chromosome constitution of 7**Ns** disomic addition lines based on mitotic and meiotic cytogenetics and GISH; b) to develop and characterize EST-SSR and EST-STS markers for 7**Ns** based on the collinearity between wheat and *P. huashanica*; c) to evaluate the leaf rust resistance and agronomic traits attributed to 7**Ns** after their addition to common wheat.

## Results

**Figure 1:**
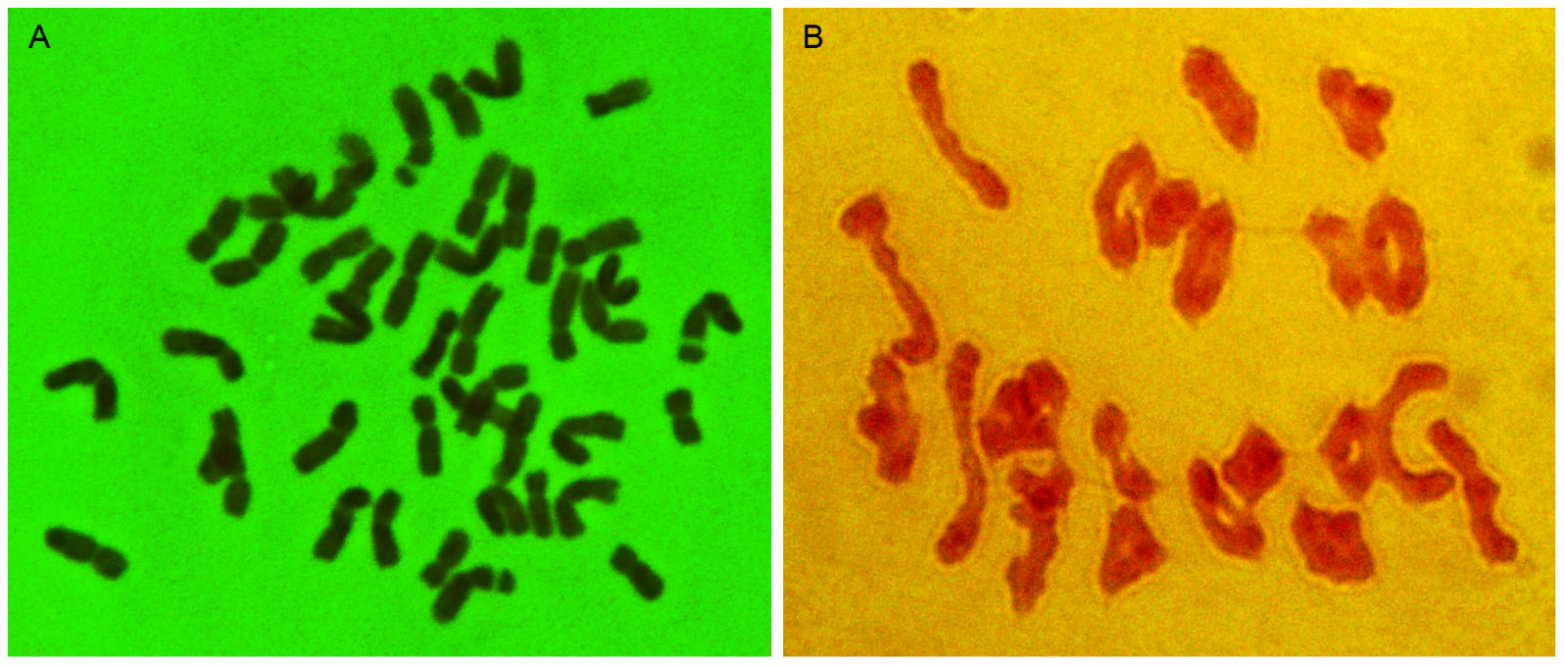
Mitotic and meiotic patterns of the wheat-*Psathyrostachys huashanica* addition line 2-1-6-3. **a** Somatic chromosomes in the root tips, 2*n* = 44. **b** Chromosome behavior of pollen mother cells during metaphase I, 2*n* = 22 II.

Development and cytological characterization of the wheat-*P. huashanica* disomic addition line A wheat-*P. huashanica* heptaploid hybrid H8911 was obtained in 1991, which was backcrossed continuously with common wheat cv. 7182, followed by one generation of selfing. Twenty 2-1-6-3 plants were shown to have a pair of alien chromosomes from *P. huashanica* according to the mitosis and meiosis analysis, and the chromosome number and configuration were 2*n* = 44 = 22 II (Figs. 1a and 1b). The chromosome pairing behavior was characterized in the pollen mother cells (PMCs) during metaphase I and 100 2-1-6-3 cells were monitored regularly to detect the average numbers of univalents (0.61), ring bivalents (17.74), and rod bivalents (3.18) (Table 1). No trivalents or quadrivalents were detected. Complete homologous chromosome pairing was observed in 92% of the PMCs. These results suggest that the wheat-*P. huashanica* disomic addition line 2-1-6-3 was cytologically stable.

**Table 1:** Chromosome pairing during metaphase I in pollen mother cells from *Psathyrostachys huashanica*, wheat cv. 7182, and the disomic addition line 2-1-6-3.

Materials	2*n*	No. of cells	Chromosome configuration				Chiasmata/cell
			Univalent	Bivalent			
				Rod	Ring	Total	
*P. huashanica*	14	50	–	2.98	4.02	7	13.89
			–	(0–5)	(2–7)	(7)	(13–14)
7182	42	50	0.08	2.92	17.43	20.35	39.78
			(0–1)	(0–4)	(14–21)	(19–21)	(39–42)
2-1-6-3	44	100	0.61	3.18	17.74	20.92	40.98
			(0–4)	(1–6)	(16–21)	(20–22)	(40–44)

–indicates no data record.

### GISH analysis

**Figure 2:**
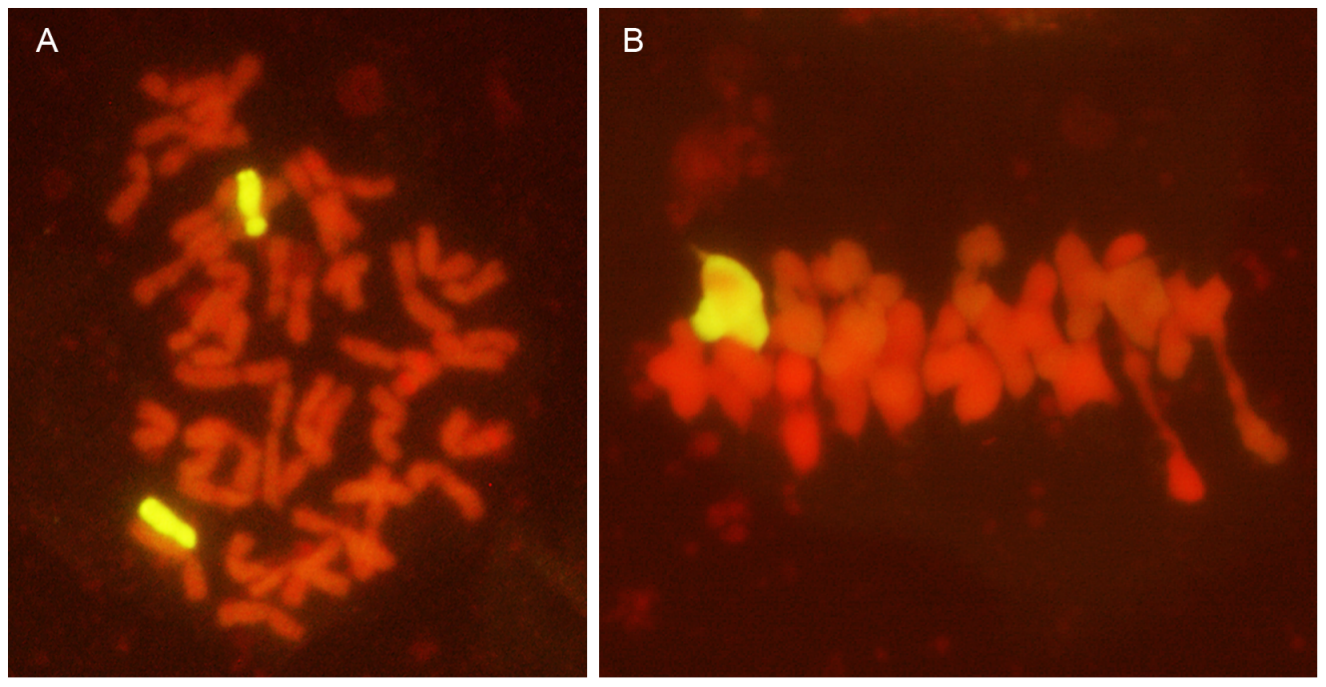
GISH analysis of the wheat-*Psathyrostachys huashanica* addition line 2-1-6-3 at mitosis and meiosis using Ns genomic DNA from *Psathyrostachys huashanica* as a probe. **a** Somatic metaphase indicating two *Psathyrostachys huashanica* chromosomes (yellowish-green color). **b** Pollen mother cells during meiotic metaphase I, showing a ring bivalent chromosome from *Psathyrostachys huashanica* (color figure online).

Mitotic and meiotic GISH were conducted using the whole genomic DNA of *P. huashanica* as a probe to monitor the chromosomal constitution of addition line 2-1-6-3. Two distinctive alien chromosomes with strong hybridization signals were detected in the root tip cells of 2-1-6-3 (Fig. 2a). Similarly, one rod bivalent was detected with a yellowish-green hybridization signal in the PMCs during metaphase I (Fig. 2b). These results suggest that 2-1-6-3 contained two alien chromosomes from *P. huashanica* and that these two chromosomes paired to form ring bivalents. Thus, the addition line 2-1-6-3 had 42 wheat chromosomes and two chromosomes from *P. huashanica*.

### Development of *P. huashanica* chromosome-specific markers

**Figure 3:**
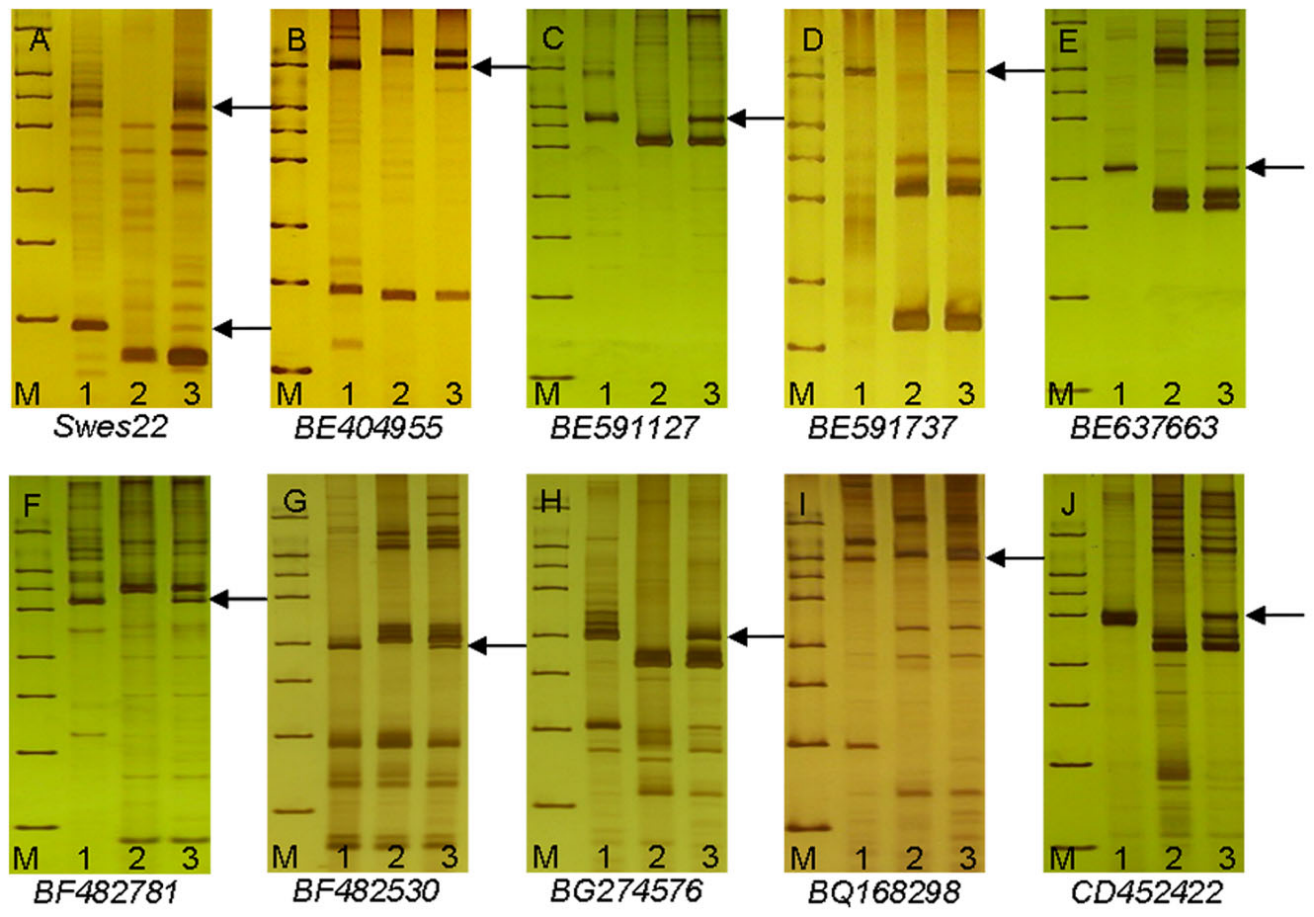
Identification of *Psathyrostachys huashanica* chromosomes using EST-SSR and EST-STS markers in disomic addition line 2-1-6-3 and its parents, wheat 7182 and *Psathyrostachys huashanica*. PCR was used to amplify specific bands in the addition line, which were attributable to chromosome 7Ns from *Psathyrostachys huashanica* (arrows). **M** marker, **1** *Psathyrostachys huashanica*, **2** 7182, **3** chromosome addition line 2-1-6-3. Arrows indicate the diagnostic amplification products for *Psathyrostachys huashanica* (a, b, c, d, e, f, g, h, i and j).

Wheat EST-SSR multi-loci markers were developed for discriminating *P. huashanica* chromosomes. After screening 55 EST-SSR markers, we obtained 20 EST-SSR primer pairs, which were polymorphic in 7182 and *P. huashanica*, and located on different wheat chromosomes. These polymorphic markers were then used to analyze the addition line 2-1-6-3. Only one primer, *Swes22*, which mapped onto chromosomes 7**A** and 7**B**, produced stable and clear polymorphic bands in 7182, *P. huashanica*, and 2-1-6-3 (Fig. 3a; Table 2).

**Table 2:** Primers used as specific markers for chromosome 7Ns in *Psathyrostachys huashanica*.

Marker	Type	EST accessionNo.^b^	Primer	Location	Annealing temperature (°C)
*Swes22* a	EST-SSR	Chen et al. (2005)	F: CTGGAAGACCAAGGAGGGA	7A 7B	55
			R: GGAAACTGGGAGGGCAAT		
BE404955	EST-STS	BE404955	F: CGTGGCATTATAGCGAGGAT	C7A 7BS C7D	60
			R: ATTGGTGAAGCAGAAGCGAT		
BE591127	EST-STS	BE591127	F: GCAGCTCATCTTCATGGTCA	7AS 7BS 7DS	60
			R: CGTTGCAGCAATCAGTCCTA		
BE591737	EST-STS	BE591737	F: TAACCGCAGCTTTCTCATCC	7AS 7BS 7DS	60
			R: AGCAGCTAGGAGGGTGTCTG		
BE637663	EST-STS	BE637663	F: ACTGTTGCTTCGCTCCAAGT	7AL 7BL 7DL	60
			R: GTTCCATTTCCGATGTGCTC		
BF482781	EST-STS	BF482781	F: CATCAGGAAGTCTAAGGCCG	7AL 7BL 7DL	60
			R: GAGAAGCAACCCAGCAACTC		
BF482530	EST-STS	BF482530	F: CAAGTACACGGTGGTGTTCG	7AL 7BL 7DL	60
			R: AAGTCCAGGTACCCTGTCCC		
BG274576	EST-STS	BG274576	F: AGATGAACTCTGCGCTGGAT	7A 7BL 7DS	60
			R: AGCTCGATGATCTGCTTGGT		
BQ168298	EST-STS	BQ168298	F: GCTCTCGCTCATCATCAACA	7AS 7BS 7DS	60
			R: CTCGCAATGGTACCAAGGTT		
CD452422	EST-STS	CD452422	F: GAAGTTCTTGAGCAGCTCCG	7AL 7BL 7DL	60
			R: TCAGATGCCTACGATGATGG		

a The marker *Swes22* was previously mapped by Chen et al (2005) and was verified in this study.

b EST accession No. in the database: http://wheat.pw.usda.gov/SNP/new/pcr_primers.shtm.

One hundred and fifty EST-STS makers from 336 pairs produced polymorphic bands in 7182 and *P. huashanica*. Nine of these 150 markers produced stable and clear polymorphic bands in addition line 2-1-6-3 and they could be used to trace the corresponding *P. huashanica* chromosomes. The primers *BE404955*, *BE591127*, *BE591737*, *BE637663*, *BF482781*, *BF482530*, *BG274576*, *BQ168298* and *CD452422*, which were located on different chromosomal arms of the seventh homoeologous group, amplified polymorphic bands in *P. huashanica* and addition line 2-1-6-3, but not in the female parent 7182 (Fig. 3b–3j; Table 2). This indicated that one pair of the *P. huashanica* chromosomes added to addition line 2-1-6-3 could be separated into the seventh homoeologous group, i.e., 7**Ns**, and one EST-SSR marker and nine EST-STS markers could be used as specific markers to detect *P. huashanica* chromosome 7**Ns** in a wheat background.

### Sequence analysis

We randomly selected five EST-STS markers (*BE404955*, *BE591127*, *BE637663*, *BF482781* and *CD452422*). The polymorphic DNA fragments of the markers from *P. huashanica* and disomic addition line 2-1-6-3 were cloned and sequenced (Fig. 3b, 3c, 3e, 3f and 3j). The sequences of those polymorphic markers were conserved to the (File S1: S1, S2, S3, S4, S5, S6, S7, S8, S9, S10). Sequence homology searches were also conducted using DNASTAR 6.0 (SeqMan). The results showed that the amplified polymorphic bands of *P. huashanica* and disomic addition line 2-1-6-3 shared 100% sequence identity, which demonstrated that *P. huashanica* chromosome 7**Ns** had been introduced into wheat.

### Leaf rust evaluation

**Figure 4:**
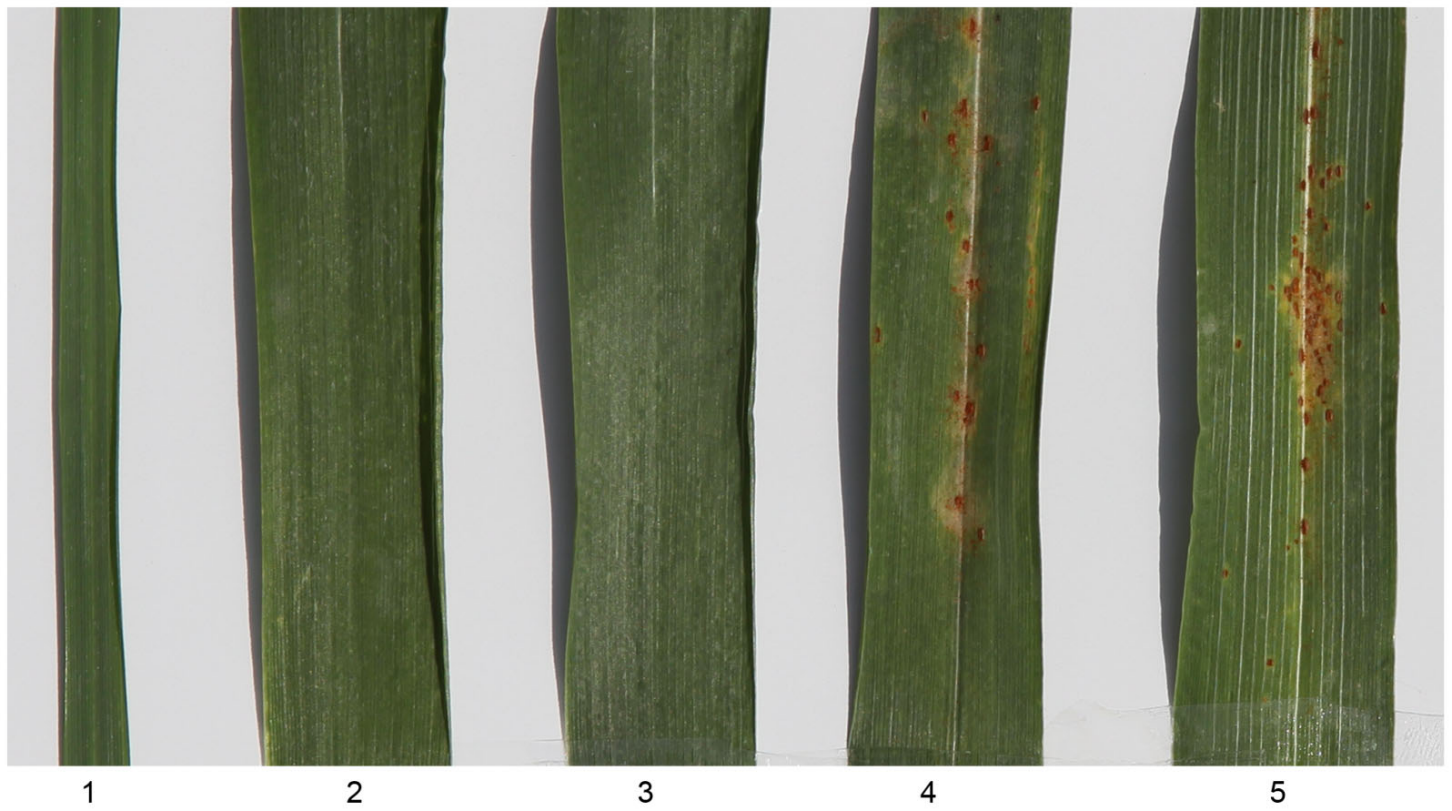
Leaf stripe reactions of the disomic addition line 2-1-6-3 and its parents, wheat 7182 and *Psathyrostachys huashanica*, and Shaan 229, during the adult plant stage using a mixture of leaf rust pathotypes. **1** *Psathyrostachys huashanica*, **2** and **3** 2-1-6-3 (resistant to leaf rust), **4** 7182, **5** Shaan 229 (susceptible to leaf rust).

We tested *P. huashanica*, 7182, 2-1-6-3, and sensitive cultivar Shaan 229 using mixed spore leaf rust cultures in the field. Fungal plaques usually appeared on the lower leaves whereas the flag leaves were rust-free until senescence when traces of leaf rust appeared. After three years of observation and testing, we found that the female parent 7182 and control cultivar Shaan 229 exhibited type 3 infection responses (see Materials and Methods), indicating their susceptibility to leaf rust. By contrast, *P. huashanica* and addition line 2-1-6-3 displayed type 0 infection responses, indicating that they were resistant to leaf rust (Fig. 4; Table 3). This suggested that disomic addition line 2-1-6-3 had inherited the leaf rust resistance gene(s) from *P. huashanica*.

**Table 3:** Agronomic traits of *Psathyrostachys huashanica*, wheat cv. 7182, disomic addition line 2-1-6-3 and leaf rust susceptible control cultivar Shaan 229.

Characters	*P. huashanica*	7182	2-1-6-3	Shaan 229
**Plant height (cm)**	80(75–85)Aa	85(80–92)Aa	75(70–81)Aa	–
**Tillering**	clump	9(6–12)Aa	15(12–18)Aa	–
**Spike length (cm)**	8(6–9)Aa	9(7–10)Aa	11(9–12)Aa	–
**Kernels per spike**	40(26–55)Bb	55(45–60)ABb	95(78–115)Aa	–
**Spikelets per spike**	15(12–18)Aa	16(12–20)Aa	20(14–24)Aa	–
**Kernels per spikelet**	3(1–4)Aa	3(2–4)Aa	5(4–6)Aa	–
**Thousand-grain weight (g)**	3.5(2.8–4.2)Bc	40(39–45)Ab	50(45–55)Aa	–
**Awn length (cm)**	0.7(0.2–1)Aa	5(1–6)Aa	5(1–7)Aa	–
**Leaf rust resistance**	0	3	0	3

The data in the column indicates mean (range) respectively.

–indicates no data recorded. Significant differences in the means are indicated at the *P*<0.01 (capital letters) and *P*<0.05 (lower-case letters) levels, based on Duncan's multiple range tests.

### Agronomic traits of disomic addition line 2-1-6-3

**Figure 5:**
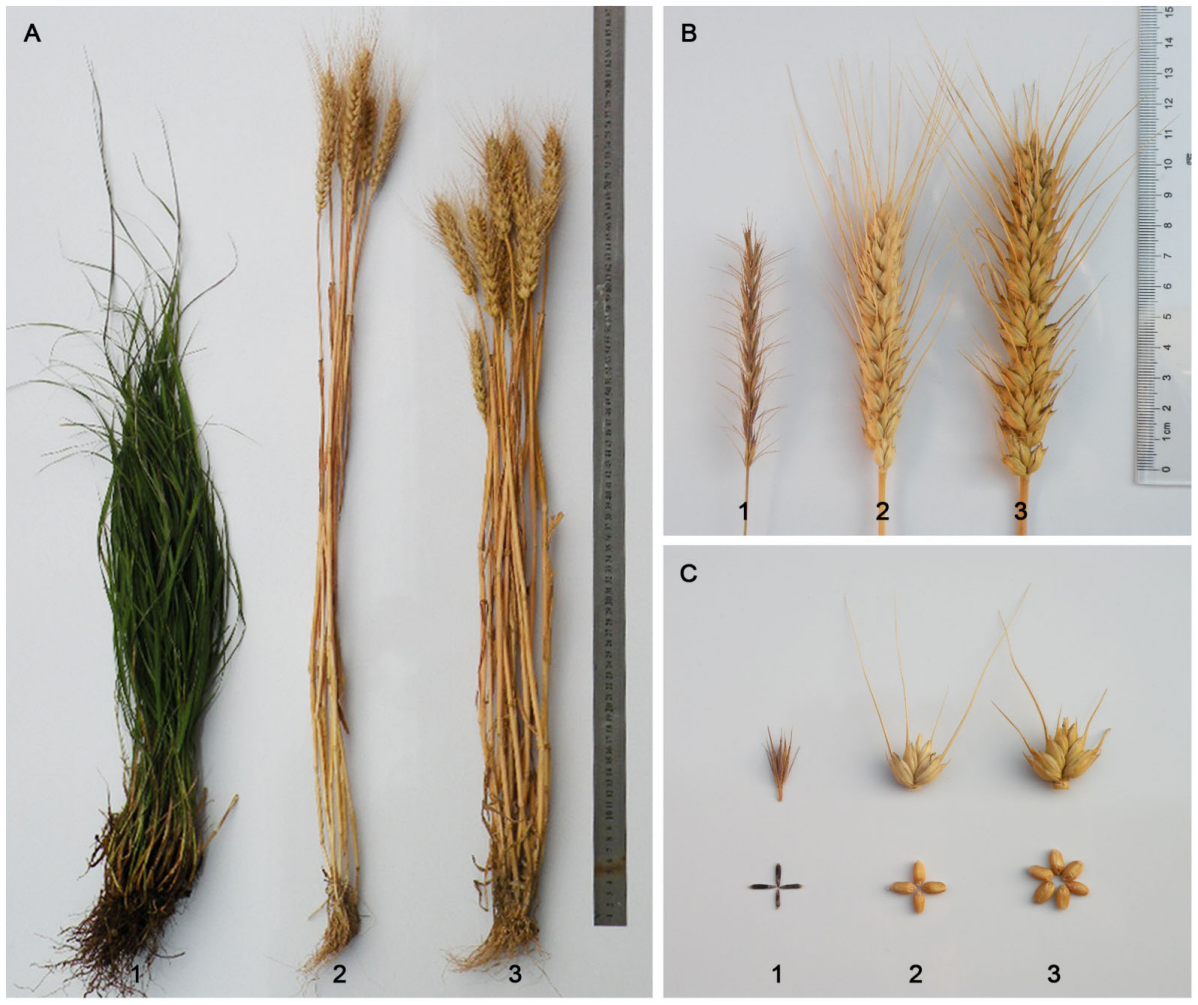
Morphological comparison of adult plants, spikes, spikelets, and seeds from disomic addition line 2-1-6-3 and its parents, wheat 7182 and *Psathyrostachys huashanica*. **a** adult plants, **b** spikes, **c** spikelets and seeds. **1** *Psathyrostachys huashanica*, **2** 7182, 3 2-1-6-3.

Disomic addition line 2-1-6-3 was tested for three years in the field and the plants appeared to be similar to wheat, except spike length, number of spikelets per spike and kernels per spikelet were greater than those of their parents, whereas the plant height was lower compared with the parents. The spikes of 2-1-6-3 plants had a full awn and they were very similar to 7182, which distinguished it from the male parent *P. huashanica* with its small tip awns. The 2-1-6-3 spikes were also bulkier and their lengths reached 11 cm, i.e., about 2 cm and 3 cm longer than the spike lengths of 7182 and *P. huashanica*, respectively. As expected, 2-1-6-3 had a number of excellent traits. In particular, there were six kernels per spikelet in the main spike. It produced plump red seeds, which were similar to those of the female parent 7182, except they had a higher 1000-kernel weight (about 50 g) (Fig. 5). According to Duncan's multiple range test (*P*<0.01 and *P*<0.05), there were significant differences between the disomic addition line 2-1-6-3 and its parents, 7182 and *P. huashanica*, in terms of the number of kernels per spike and thousand-grain weight (Table 3).

## Discussion

After successfully transferring an alien chromosome(s) or chromosome segment(s) into wheat, the next step is to identify the actual alien chromatin [3]. GISH is an efficient and accurate tool for tracking alien chromosomes or smaller fragments in a wheat background [25]. Therefore, it was employed to identify the chromosomal configuration and composition of the 2-1-6-3 line, which had a superior numbers of florets and grains per spike. During the mitotic and meiotic phases, *P. huashanica* was used as a probe and Chinese Spring as a blocker. To demonstrate that genes have been incorporated from a wild species into wheat, the most important prerequisite is detect normal meiotic pairing and recombination between corresponding genomes in the F_1_ hybrids and in their subsequent selfed and backcrossed progeny [26]. The cytogenetic observations and GISH analysis of 2-1-6-3 confirmed its cytogenetic stability and its chromosomal composition was 2*n* = 44 (Figs. 1 and 2). Two chromosomes from *P. huashanica* were detected during meiotic metaphase I in PMCs and they formed bivalents with no lagging in the equatorial plate (Fig. 2b). This showed that the *P. huashanica* chromosomes could engage in normal synapsis, pairing, and separation in a wheat background, which could facilitate the expression of its excellent agronomic traits in correlated homologous groups. Indeed, the production of alien addition lines is an important step during the successful transfer of genes into wheat because it plays a bridging role in the creation of alien substitution lines and translocation lines.

The transferability of EST-based PCR markers among related species allows introduced alien chromatin to be tracked easily [27], [28]. EST-SSR is recognized as an efficient and stable method for developing chromosomal molecular markers based on their association with conserved expressed sequences [29]. EST sequences derived from gene transcripts are likely to be conserved in wheat and related species so they are used widely for identifying homoeologous relationships between wheat chromosomes and those of aliens, including *Secale cereale* L. [30], *Aegilops tauschii*. [31], *Dasypyrum villosum* [32], *Hordeum chilense* [33], and *Thinopyrum ponticum* [8], [34]. To further confirm the transfer of chromosomes from *P. huashanica*, we screened 55 EST-SSR and 336 EST-STS multiple-loci pair primers from wheat. One EST-SSR and nine EST-STS markers were tested successfully in the wheat-*P. huashanica* 7**Ns** disomic addition line. One EST-SSR marker, *Swes22*, which was located on wheat chromosomes 7**A** and 7**B**, specifically amplified polymorphic bands in *P. huashanica* and 2-1-6-3 (Fig. 3a). This indicated that the addition line possessed a wheat background but it also expressed unique bands from *P. huashanica*, which was supported by the cytogenetic analysis and GISH results (Figs. 1 and 2). Nine EST-STS makers, which mapped onto the short arm, long arm, and centromere of wheat homoeologous group 7, produced specific bands in 2-1-6-3 and the alien parent (Fig. 3b–3j). These results demonstrated that the chromosome from *P. huashanica* had a homoeologous relationship with the seventh group. The makers identified here could also be used as a rapid and direct method for screening progeny lines containing 7**Ns**. These *P. huashanica*-specific chromosome markers will be useful for the accurate and rapid screening of large populations of alien chromosome lines during wheat breeding programs. These markers could also be employed for comparative gene mapping, chromosomal evolutionary analysis, and gene introgression during wheat improvement using *P. huashanica* accessions as gene donors.

The wild relatives of wheat are reservoirs of genetic variability for various traits, including resistance to rust diseases [35]. Over 17 known *Lr* genes have been introgressed into common wheat from *Aegilops* (goatgrass) [3]. Similarly, four leaf rust resistance genes were transferred from *Agropyron*, i.e., *Lr19*, *Lr24*, *Lr29*, and *Lr38* [4], while five *Pr* genes and three *Lr* genes were derived from cultivated rye (*Secale cereale* L.) [4], [36], [37]. A previous study indicated that leaf rust resistance genes were closely linked to homoeologous group 7, i.e., the leaf, stem, and stripe rust resistant line (Z4) carried an additional group 7 chromosome from *Thinopyrum intermedium* [38]. Wheat-*Hordeum* addition lines possessed common bunt and septoria leaf blotch resistance, which was conferred by gene(s) on chromosome 7 in field experiments [39], [40]. Chromosome 7**E** from *Lophopyrum ponticum* also carries a valuable leaf rust resistance gene designated *Lr19* [41]. In the present study, we demonstrated the transfer of putative new leaf rust resistance gene(s) from a wild species, *P. huashanica*, to common wheat by the development of the 7**Ns** chromosome addition line 2-1-6-3. This novel addition line will provide a solid foundation for isolating resistance gene(s) from 7**Ns**, as well as for developing highly specific diagnostic markers. This is the first time that a progeny line from *P. huashanica* has shown resistance to leaf rust and it will provide a new resource for the development of rust resistance in current breeding programs, eventually leading to the development of resistant cultivars.

In this study, the presence of *P. huashanica* chromosome 7**Ns** demonstrated that homoeologous group 7 was associated with yield enhancement, particularly high numbers of kernels per spike and a higher thousand-grain weight. It has also been reported that the incorporation of a 7**DL**/7**Ag** translocation from *Agropyron elongatum* (Host) Beauv. caused a significant increase (9%) in biomass at harvest during non-moisture stress trials [42]. The 7**DL**.7**Ag** translocation line from *Lophopyrum elongatum* carried *Lr19*, a leaf rust resistance gene, and it was found to be associated with a significant increase in the grain yield in irrigated, disease-free conditions [43]. The results described in this previous study demonstrated that the introgression of chromosome 7**Ag** increased the grain yield and it also carried a leaf rust resistance gene, *Lr19*, which matched the results obtained using our *P. huashanica* 7**Ns** disomic addition line 2-1-6-3. The superior spike trait of addition line 2-1-6-3 will be particularly significant for enhancing the crop yield because it allows more kernels to be formed per spike. Duncan's multiple range test showed that the thousand-grain weight differed significantly between 7182 and 2-1-6-3 (Table 3). This suggests that addition line 2-1-6-3 confers resistance to leaf rust but it may also increase the wheat yield. Therefore, our study showed that new gene(s) located on 7**Ns** of *P. huashanica* and introduced into a wheat background increased the leaf rust resistance during the adult age and also enhanced the potential yield. In addition, this study also provides novel insights into the utilization of homoeologous group seven chromosomes by *P. huashanica* in a wheat background.

## Materials and Methods

### Plant material

Common wheat cv. 7182 (2*n* = 42, **AABBDD**), *P. huashanica* (2*n* = 14, **NsNs**), and the progeny of 2-1-6-3 (2*n* = 44) were used in this study. We selected the disomic addition line 2-1-6-3 (BC_2_F_2_), which had a chromosome number of 2*n* = 44, a high thousand-grain weight, a superior numbers of florets and grains per spike, and resistance to leaf rust. The parental wheat cultivar 7182 and *P. huashanica* were included as controls to assess their agronomic traits and leaf rust resistance, and were used in the EST-SSR and EST-STS analysis. Wheat cv. Shaan 229 was used as a susceptible control in the leaf rust disease response tests. Chinese Spring was used as a source of blocking DNA during the GISH analysis. These plant materials are deposited at the Shaanxi Key Laboratory of Genetic Engineering for Plant Breeding, College of Agronomy, Northwest A&F University, Shaanxi, China.

### Ethics statement

The plant collection of *Psathyrostachys huashanica* Keng was approved in 1987 by the Wildlife Conservation and Nature Reserve Management Office of Shaanxi Province, China. The field studies were also permitted by Laboratory Management, Northwest A&F University. The study was carried out in strict accordance with the regulation of the Shaanxi Key Laboratory of Genetic Engineering for Plant Breeding, College of Agronomy, Northwest A&F University.

### Cytogenetic analysis

Seeds were germinated in the dark at 23°C until the roots reached 1–2 cm. The roots were cut and incubated in ice-cold water overnight, and then they were fixed in Carnoy's solution with 95% ethanol-acetic acid (3∶1, v/v). PMCs were collected from young panicles and fixed in absolute ethanol-chloroform-glacial acetic acid (6∶3∶1, v/v). Mitotic and meiotic chromosomes were squashed on a slide in a drop of acetocarmine and 45% acetic acid, before they were used for cytological observations and GISH, respectively. The cover slips were removed from the GISH slides after freezing with liquid nitrogen, followed by air drying and storage at −20°C.

### GISH

The total genomic DNA were extracted from the fresh leaves of *P. huashanica* using the improved CTAB method [44]. GISH was performed using a published method [45] with a minor modification. The *P. huashanica* DNA probe was labeled with digoxigenin (digoxigenin-11-dUTP, DIG; Roche, Germany) via the nick translation method. A total of 40 µl hybridization solution was overlaid on a slide, which contained 4 µl 20× SSC, 1 µl ssDNA (salmon sperm DNA 5 µg/µl), 1 µl 10% (W/V) SDS (sodium dodecyl sulphate), 8 µl 50% (W/V) dextran sulfate, 20 µl deionized formamide, and 100 ng probe DNA, and it was made up to 40 µl using autoclaved deionized water. Denaturation was conducted at 95°C for 10 min. The hybridization conditions were 80°C for 5 min and 37°C for 16 h using a hybrite system (ThermoBrite, USA). Next, 50 µl of FITC with Anti-dig antibody was added to detect and visualize the labeled chromosomes. Fluorescence signals were viewed and photographed using a microscope (Olympus BX60) with a Photometrics SenSys CCD camera.

### EST-SSR and EST-STS analysis

EST-SSR and EST-STS markers were used to determine the homoeologous relationships among the added *P. huashanica* chromosomes. Genomic DNA was isolated from the wheat-*P. huashanica* addition line and both parents, as previously described [44]. To characterize the genomic composition of the wheat-*P. huashanica* addition lines, we used 55 EST-SSR and 336 EST-STS multiple-loci primer pairs (based on published sources [46], [47], [48] and http://wheat.pw.usda.gov/SNP/new/pcr_primers.shtml), which were distributed evenly among seven wheat homoeologous groups. PCR amplification was conducted in a 20 µl reaction mixture that contained 2 µl 10× PCR buffer, 2 µl primer (2.5 µmol/ml), 2 µl DNA template (40–60 ng/µl), 1.6 µl dNTPs (2.5 µmol/ml), 1.6 µl MgCl_2_ (2.5 mmol/ml), 0.1 µl *Taq* polymerase (5 U/µl), and 10.7 µl ddH_2_O. The amplification procedure was as follows: initial denaturation for 3 min at 94°C, followed by 35 cycles of 1 min at 94°C, 50 s at 60°C, 1 min at 72°C, and a final extension for 10 min at 72°C. The PCR products were separated in standard conditions using 8% non-denatured PAGE gels, which were visualized by silver staining.

### Cloning and sequencing of the EST-STS product

The putative PCR bands were excised from the 8% non-denatured PAGE gels and extracted using a gel extraction kit. The recovered DNA fragments were cloned into the pMD19-T vector and transformed into *Escherichia coli* DH5a-competent cells by heat shock transformation. Positive colonies were determined by blue/white screening. The white colonies were picked from LB-ampicillin plates and the recombinant DNA was extracted from each overnight cultured colony using a plasmid kit. DNA sequencing was performed at Sangon Biotech (Shanghai, China). Sequence homology searches were performed using DNASTAR 6.0 (SeqMan).

### Leaf rust and morphological evaluation

Adult plant reactions to leaf rust were determined during the 2009, 2010, and 2011 field growing seasons in Yangling, Shaanxi, China (N 34°16′56.24″, E 108°4′27.95″), which has a relatively warm climate, abundant rainfall, constant sun, and rich soil. The plants were arranged separately in a completely randomized block design using two replicates and artificial inoculations were conducted on several occasions by dusting a mixture of leaf rust pathotypes (FHTT, PHST, and FHST) evenly over leaves until the susceptible checks were fully infected. The inoculation procedure and evaluations of reactions followed published methods [49]. Plants that presented response types 0, 1, 2, and X were considered to be resistant (R), whereas plants with type 3 and 4 responses were sensitive (S).

During the 2009, 2010, and 2011 sowing seasons, completely randomized block designs with two replicates were used to evaluate all of the traits of 7182, *P. huashanica* and the disomic addition line 2-1-6-3, i.e., plant height, tillering, spike length, kernels per spike, spikelets per spike, kernels per spikelet, thousand-grain weight, and awn length. After harvesting, 20 plants were examined in each plot to assess each trait and they were compared using Duncan's multiple range test (*P*<0.01 and *P*<0.05), which was conducted using the General Linear Model procedure in the SAS package (version 9, SAS Institute Inc., Cary, NC, USA). All of the parameters were means and they were compared with their parental species.

## Supporting Information

File S1 **Sequences.**
(TXT)
